# Reliability of a Novel Grid-Based Dental Rubric as an Effective Assessment Tool for Evaluating Class II Tooth Preparations in Preclinical Settings

**DOI:** 10.7759/cureus.105921

**Published:** 2026-03-26

**Authors:** Rhythm Bains, Munira Hirkani, Praveen Iyer, Vivek K Bains, Nishi Singh

**Affiliations:** 1 Conservative Dentistry and Endodontics, King George's Medical University, Lucknow, IND; 2 Physiology and Medical Education, Seth GS Medical College and King Edward Memorial Hospital, Mumbai, IND; 3 Anatomy and Medical Education, Seth GS Medical College and King Edward Memorial Hospital, Mumbai, IND; 4 Periodontology, Saraswati Dental College and Hospital, Lucknow, IND

**Keywords:** assessment, bias, consistency, dental, reliability, rubrics, validity

## Abstract

Introduction

Assessment of preclinical operative skills is often limited by subjectivity and inconsistent grading. Traditional glance-and-grade methods provide minimal formative feedback. Criteria-based rubrics may improve objectivity, reliability, and learning outcomes in preclinical dental education. The aim of the present study was to test the reliability of a novel, criteria-weightage-based rubric for the evaluation of Class II restorations in preclinical settings.

Methods

A purposive sample of 10 subject-matter experts and 10 non-subject-matter experts was selected, and the rubric document was shared with them. Face Validity Index (FVI) and Content Validity Index (CVI) were calculated. The validated rubric was pilot-tested to evaluate preclinical competency in Class II cavity preparation (N = 36 BDS second-year students). Agreement between scores of two sets of evaluators using the traditional and rubric methods was used to measure inter-examiner reliability (consistency), and agreement between scores of the same examiners evaluating the same preparation after a seven-day washout period was an indicator of intra-examiner reliability (reproducibility).

Results

The rubric exhibited an adequate FVI of 0.96 and CVI of 0.98. The rubric's inter-examiner reliability and intra-examiner reliability were significantly higher than those of traditional methods. For the rubric method, the intraclass correlation coefficient (ICC) showed moderate agreement (ICC = 0.526), while the Pearson correlation coefficient indicated a statistically significant positive correlation between observers (r = 0.584, p < 0.001). Overall, the rubric-based method produced consistently higher ICC values for intra-examiner reliability (0.573-0.600) compared with the traditional method (0.265-0.396).

Conclusion

Based on the findings of the pilot study, the rubric-based assessment method is recommended as the primary tool for evaluating preclinical cavity preparations, as it demonstrated comparatively better inter- and intra-observer reliability than the traditional glance-and-grade approach.

## Introduction

Assessment and evaluation are an integral and crucial step of any learning method. A good assessment method should not only serve as a source of information about the scores attained by a student, but also be able to reflect on the possible areas of weakness and highlight the areas where the student has performed well. Additionally, it should serve as an indicator for the instructor or mentor about the specific areas where a larger number of students are scoring poorly, thus suggesting that an improvement in instruction is required for that particular segment of the course [1,2].

Dental education encompasses not only the theoretical aspects of dental disease and its management, but also significant practical and hands-on learning experiences. Undergraduate dental students receive early clinical exposure and begin working with patients in their third year of the curriculum [3,4]. Considering the responsibility of dental graduates for interacting with the hard and soft tissue structures of the oral and maxillofacial regions, the importance of quality dental education cannot be underestimated [5].

Procedures performed by dental undergraduates, such as tooth preparations, restorations, endodontic treatment, prosthesis fabrication, and dental extractions, require them to deal with intricate hard and soft tissues of the maxillofacial region, as well as a thorough understanding of the anatomy and morphology, the clinical behaviour of bioactive materials, and the correct techniques to carry out these procedures [6]. Students are continuously evaluated for these clinical competencies through both formative and summative assessments. A reliable method for evaluating these clinical competencies during the training period is a crucial step in strengthening the education system [7]. Presently, most dental schools use the traditional glance-and-grade method to evaluate various clinical competencies, which does not give students the opportunity to understand their strengths or weaknesses. Furthermore, traditional methods of assessment are inherently associated with an element of subjectivity and bias [8].

Assessment methods, such as the Objective Structured Clinical Examination (OSCE) and the Objective Structured Practical Examination (OSPE), overcome the problem of subjectivity to some degree. Still, they do not provide strength/weakness analysis feedback to the student, as they do not measure the achievement level against a particular competency component [9]. Recently, various dental educators have proposed rubrics as a method of evaluating undergraduate clinical dental competencies, such as crown preparations in orthodontics or pediatric restorative procedures [10,11]. Still, an analytical rubric for assessing Class II dental restorations in preclinical conservative dentistry settings has not been established. A rubric is a scaled tool with levels of achievement and clearly defined criteria related to each level, placed in a grid. Rubrics differ from simple checklists and rating scales in that they include descriptions of each criterion for each level of performance [12].

In dental education, subjectivity is unavoidable when students perform procedures in the preclinical or clinical environment, or present a case orally or in writing. A rubric specifies teaching and learning outcomes for both teacher and student, thereby reducing the subjectivity inherent in these assessments. Rubrics have been proposed for use in both formative and summative assessments. Formative assessment generally provides feedback to the learner, with suggestions for improvement, while summative evaluation typically involves some judgment of student progress. Keeping in mind these challenges with traditional methods, a rubric was developed for the assessment of Class II tooth preparations (GV Black's classification) [13], prepared by BDS II-year students on typodont teeth, in preclinical settings.

With this background, the present study aimed to evaluate the face validity, content validity, and reliability of the novel criteria-based rubric as an assessment method for Class II tooth preparation in preclinical settings. 

## Materials and methods

Development of the rubric

The development of the rubric was completed in three phases, correlating with the need, conceptualization, and the tool's validity. The first stage of developing the tool was a qualitative phase, where semi-structured interviews were conducted with a panel consisting of subject experts (with a teaching experience of at least 10 years in the subject of restorative dentistry) and experts with expertise in medical education and curriculum planning. In the second stage, based on experts' opinions and an extensive review of credible scientific references relevant to the research subject, a 12-item list was compiled. Each item of the assessment tool had three components: a well-defined criterion, the weightage associated with that criterion, and three defined levels of achievement (Table 1).

**Table 1:** Validated rubric for the assessment of Class II (GV Black's classification) amalgam restorations in preclinical settings Rubric prepared to assess restoration of Class II cavities (GV Black's classification) [13].

Competency Weightage		Competent (Full = 2)	Needs Improvement (Half = 1)	Error (0)	Max. Score
Instrument tray set up, Weightage: 0.5	The instrument tray should be neatly presented, with proper table coverage, such as a McIntosh sheet or green cloth.	The tray setup is neat/ clean. Has all instruments related to diagnosis, tooth preparation, restoration, and finishing (All types of explorers, condensers, carvers, and retainers), and also has a McIntosh sheet.	The tray setup typically includes most instruments, with at least one of each type (explorer, condenser, carver, or retainer).	The tray is not set properly or is not clean. Many of the instruments are missing. The Table McIntosh cover sheet is also missing.	1
Tooth preparation outline, Weightage: 2	Maxillary molar	The preparation outline preserves the cusps, oblique ridge & marginal ridges (1.6 mm). The preparation width doesn’t exceed one-fourth of the intercuspal distance. The reverse curve is included in the preparation.	The preparation outline preserves the cusps, oblique ridge & marginal ridges. BUT The preparation width exceeds one-fourth of the inter-cuspal distance, but doesn’t exceed one-third of the inter-cuspal distance. The reverse curve is missing.	The preparation encroaches on the oblique ridge and the cusps. The marginal ridges are preserved less than 1 mm. Cavity width exceeds more than one-third of the inter-cuspal distance.	4
	Mandibular molar	The preparation outline preserves the cusps, oblique ridge & marginal ridges. The preparation width doesn’t exceed one-fourth of the intercuspal distance. The preparation is made keeping in mind the lingual tilt of the mandibular molar.	The preparation outline preserves the cusps, oblique ridge, and marginal ridges, but the preparation width exceeds one-fourth of the intercuspal distance (but does not exceed one-third).	-	4
Tooth preparation depth, Weightage: 2	The preparation follows an ideal initial depth.	The initial depth of the preparation is limited to 1.5-2 mm when measured from the central fossa	The initial depth is more than 2 mm, but the base application can salvage it. The initial depth is less than 1.5 mm; there is scope for correcting to the desired depth.	The depth of preparation exceeds 3 mm.	4
Retention & resistance features, Weightage: 2	The preparation must incorporate features for retention & resistance form.	Resistance: The pulpal floor is flat, with a depth of at least 1.5 mm, and line angles are rounded. The 90-degree cavosurface margin is maintained, marginal ridges are preserved (>1.6 mm), and the oblique ridge is preserved (for maxillary molars). Retention: Occlusally converging buccal and lingual walls, undercuts where required, and a dovetail in the occlusal step.	All features incorporated, EXCEPT the floor is not perfectly flat, but there is scope for improvement without risking exceeding the ideal depth. Preparation encroaches on marginal ridges (<1.6 mm).	The floor is not flat, and cannot be improved without exceeding the ideal depth. Line angles are sharp. The marginal ridge thickness is excessively compromised (less than 1 mm for molars).	4
Marginal configuration, Weightage: 2	The preparation margins should have a butt joint (cavosurface configuration of 90 degrees) and a lap sliding fit of 30 degrees on the gingival seat.	The walls are parallel to the configuration of enamel rods, 90-degree cavosurface margin, convergent towards occlusal, with no unsupported enamel rods, and enamel rods resting on sound dentin. No irregularities or sharp points on the external walls.	90-degree cavosurface margin present, but the walls are irregular.	The cavosurface margin is either very acute or obtuse. The walls are irregular	4
Base material mixing & application, Weightage: 1	The candidate should mix the base material to a proper consistency.	Uses a paper pad and a plastic spatula for mixing. Mix the powder & liquid in increments to a thick consistency. The base is placed in a cavity with a thickness of 0.5mm, and gives the pulpal floor a flat form.	The final consistency is less thick than desired, but manageable. The student uses a steel spatula instead of a plastic one.	The final consistency is slurry and not suitable for the base, or the final consistency is dry and cannot be manipulated. The base material is irregularly placed, the pulpal floor is not flat after base application, and it compromises resistance form.	2
Application of matrix band/retainer/wedge, Weightage: 1	The retainer, band, and wedge should be correctly placed for proper contact development.	A Tofflemire retainer is placed along the vestibule, with the gingival slot facing gingivally. The band is tightly placed, allowing for overfilling of the initially condensed amalgam. The wedge is placed at a right angle in the interproximal embrasure, which is slightly larger in size.	Tofflemire is not as tight, which may lead to over-contoured restoration.	Tofflemire's gingival slot was placed incorrectly. The Tofflemire band is placed perpendicular/wrongly directed to the vestibule. The wedge is missing/loosely placed.	2
Restorative material mixing, Weightage: 2	The alloy and mercury are correctly dispensed and mixed (triturated).	The alloy and mercury are dispensed in a proper ratio. They are correctly mixed, leading to a homogenous mass. The excess mercury is removed by milling.	The final mix is not very homogeneous, but has a scope for improvement by further mixing and mulling.	The final blend is too shiny or too grainy and cannot be improved by further manipulation.	4
Restorative material placement, Weightage: 2	The mixed amalgam is carried to the preparation site using an amalgam carrier and is condensed there.	The material is placed in increments, compactly filling all corners of the preparation. The material is overfilled (up to 1 mm), allowing for carving. Use of correctly sized and shaped amalgam condensers.	The restoration is not adequately compacted, but has a scope for improvement.	The restoration is underfilled. The condensation is not compact. An excessive amount of unreacted mercury is visible on the occlusal surface of the restoration.	4
Finishing/polishing, Weightage: 1	The final restoration should be adequately finished & polished.	The final restoration should be smooth, shiny, and sealed at the margins.	-	The final restoration has a rough, dull appearance and has no scope for improvement.	2
Handling of amalgam scrap/biomedical waste, Weightage: 0.5	The amalgam scrap and unused mercury should be handled according to biomedical waste management guidelines.	The students are aware of the correct method of disposal and dispose of it accordingly. The amalgam scrap & mercury is disposed of in a properly labeled, covered bottle filled with water.	The students are aware of the correct method of disposal when asked orally, but they do not dispose of it correctly.	The student neither possesses knowledge nor practices it correctly. The amalgam scrap/mercury can be seen scattered on the work station.	1

In the third stage, the face and content validity of the tool were calculated. The objectives of the study were to develop and pilot-test a rubric-based assessment tool for Class II cavity preparation in a preclinical typodont setting and to evaluate its effectiveness as an objective evaluation method. Specifically, the study aimed to assess the inter-examiner and intra-examiner reliability of the rubric-based assessment in comparison with the conventional glance-and-grade method and to establish the content and face validity of the rubric for assessing students’ cavity preparation skills.

Calculation of validity

Face validity is defined as the extent to which test respondents view the test as clear and the items of the test as relevant. Few researchers suggest that raters for face validity can be either the respondents taking the test, the general public, or nonprofessional users involved in handling the test results. Generally, content validity is calculated first, followed by face validity, also known as response process validity. According to data available from previous research, the number of respondents used to calculate validity was kept at ten. These indices for face validity and content validity were calculated using freely available formulae, utilizing methodological statistics derived from expert judgment and simple proportion-based calculations, and do not require any license or proprietary permission. In this manuscript, the Face Validity Index (FVI) and Content Validity Index (CVI) were calculated based on earlier work reported by Yusoff [14,15].

Content Validity Calculation

The sheet was shared with 10 subject-matter experts (six internal and four external) via email and WhatsApp. The experts scored each item according to its essentiality on a scale of 1 to 4 (1 = item not related to the domain, 2 = item somewhat related to the domain, 3 = item quite relevant to the domain, 4 = item highly relevant to the domain).

For the CVI calculation, the formula applied was the content validity ratio (CVR): $\mathrm{CVR} = \frac{n_E - \frac{N}{2}}{\frac{N}{2}}$, where nE is the number of panelists indicating “essential” and N is the total number of panel members. CVI was calculated as: $\text{CVR of all items} / \text{total number of items}$.

Item-level CVI (I-CVI) was calculated as: $(\text{agreed items}) / (\text{number of experts})$ and scale-level CVI (S-CVI/Ave) was calculated as: $(\text{sum of I-CVI scores}) / (\text{number of items})$.

It was observed that three raters rated item 12 as 2, and two raters rated it as 1. Thus, this particular item was replaced in the rubric. Additionally, it was noted that four raters had rated item 2 as 3; therefore, this item was modified. The modified rubric was again sent to the same experts, and the final CVI was recalculated in the second round. 

Face Validity Calculation

In this study, both qualitative and quantitative methods were employed to assess the face validity of the instrument. The sheet was shared with 10 non-subject-matter experts (faculty from other departments and students), who evaluated the instrument in terms of clarity and comprehension of the items and dimensions, using wording that reflected the concept in each item.

For the qualitative method, the panelists shared their comments in the comment box after each item. For the quantitative assessment, the panelists scored each item based on clarity and comprehension on a scale of 1 to 4 (1 = the item is not clear and understandable; 2 = the item is somewhat clear and understandable; 3 = the item is clear and understandable; 4 = the item is apparent and understandable).

Items for which scores of 3 and 4 were given were marked as 1, whereas scores of 1 and 2 were marked as 0. The face validity was calculated according to the formula: $\mathrm{S-FVI/Ave} = (\text{sum of I-FVI scores}) / (\text{number of items})$.

A copyright was attained from the Copyright Office, Government of India (Registration no. L-145920/2024), for the final criteria-weightage-based rubric titled "Rubric (criteria-based grid) for assessment of competency for ideal Class II dental restorations in preclinical settings" [16].

Pilot implementation of the validated rubric

**Figure 1:**
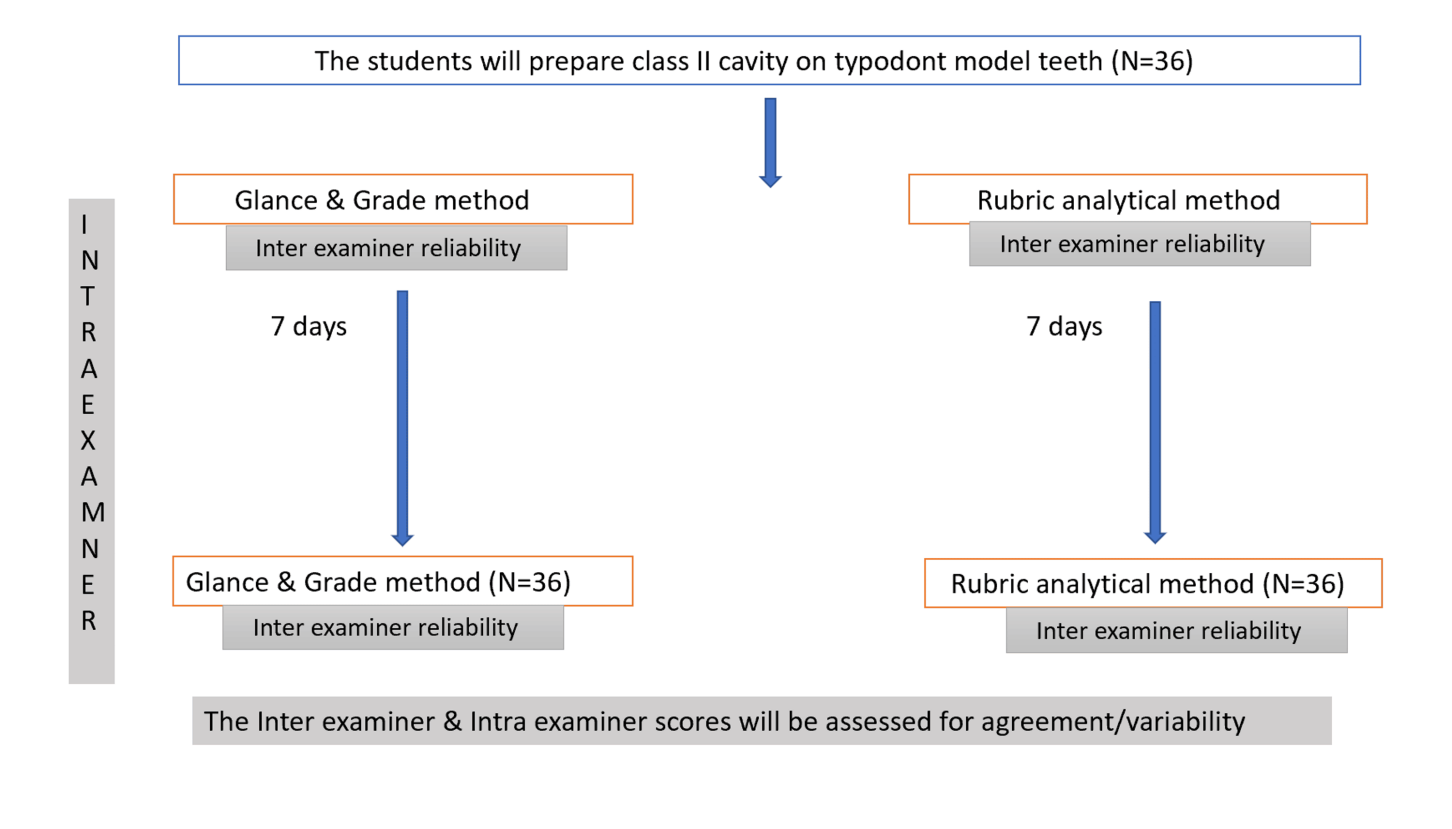
Flowchart depicting methodology of the study Class II refers to G.V. Black's Class II cavity preparation [13]. Glance-and-Grade refers to the traditional method of assessment [11]. The Rubrics method refers to assessment using the newly developed rubric [16].

Before the commencement of pilot testing, approval was obtained from the Institutional Ethics Committee of King George's Medical University (Ref. No. 148th ECMIIA/P22). The rubric was pilot-tested on second-year BDS students (N = 36), who were undergoing training in preclinical Class II cavity preparation on typodont teeth. For the purpose of the pilot testing, the students were evaluated only up to the cavity preparation stage. The reliability of the rubric was assessed by determining both inter-examiner and intra-examiner agreement among the evaluators (Figure 1).

The students and faculty involved in the pilot testing of the rubric were first sensitized to the new assessment method. Prior to commencing tooth preparation, each student’s typodont tooth was marked with a unique serial number using a permanent marker pen for identification. The typodont teeth were identified only by serial numbers to ensure the anonymity of the students. Examiners were blinded to the identity of the students and to the scores assigned by other examiners. To maintain uniformity in the exercise, all students were required to prepare a Class II cavity according to the G.V. Black Classification of Dental Caries on the first mandibular molar typodont tooth.

A total of four examiners were recruited for this purpose. All examiners had a minimum of five years of teaching experience following completion of their MDS degree. Prior to the pilot testing, both students and faculty were sensitized to the rubric-based assessment method. The examiners underwent a calibration session, in which the rubric domains and scoring criteria were explained and discussed to ensure uniform interpretation of the scoring guidelines. Sample cavity preparations were reviewed collectively during this session to establish consensus on scoring standards.

The examiners were divided into two pairs, with each pair consisting of two examiners. Examiners 1 and 2 evaluated all 36 students using the traditional glance-and-grade method, whereas Examiners 3 and 4 assessed the same students using the rubric-based assessment method. After completion of the procedure, the typodont teeth were collected for evaluation of the preparations.

The rubric consisted of predefined domains related to key aspects of cavity preparation, such as outline form, depth, wall configuration, marginal ridge preservation, and proximal box preparation.

Each domain was evaluated using a three-level performance scale: 0 = Error (Unsatisfactory); 1 = Needs improvement (Partially satisfactory); 2 = Competent (Satisfactory).

Each domain carried equal weightage. The score was calculated as the level of performance of the particular domain multiplied by the weightage. The total rubric score was calculated as the sum of the scores across all domains, with a maximum possible score of 15 points. To facilitate comparison with the traditional assessment method, the scores were later converted to percentages.

Inter-examiner agreement in the scores assigned by the two examiners within each pair was analyzed. For reliability testing, the teeth were stored after the first scoring session and re-presented to the examiners after a seven-day washout period. Before the second evaluation, the samples were re-labeled to prevent recognition of the sequence and to minimize recall bias. Examiners were not given access to their previous scores during the second evaluation. The re-labeled but same typodont teeth were re-examined by all four examiners after a seven-day washout period using the same evaluation criteria and methodology. This process helped establish the intra-examiner consistency of the assessments (Figure 1).

Statistical analysis was performed to evaluate agreement, reliability, and differences between the two assessment methods. The Pearson Correlation Coefficient (r) was used to determine the linear relationship between paired scores assigned by the examiners. Differences between paired observations were analyzed using the Wilcoxon Signed-Rank Test, a non-parametric test suitable for related samples. Inter-examiner reliability and absolute agreement were quantified using the intraclass correlation coefficient (ICC 2,1), interpreted as poor (<0.5), moderate (0.5-0.75), good (0.75-0.9), and excellent (>0.9) reliability. Agreement between paired measurements was further assessed using Bland-Altman analysis, which reports the mean systematic bias and the limits of agreement (±1.96 SD). Additionally, the Mann-Whitney U Test was used to compare percentage scores between the conventional and rubric-based assessment methods.

## Results

The FVI score was 0.96 (Table 2), and the final CVI was 0.98 (Table 3).

**Table 2:** Face validity calculation S-FVI/Ave = 0.90; UA = 0.66; S-FVI/UA = 0.96 RA: Rater’s Agreement; UA: Universal Agreement; FVI: Face Validity Index; S-FVI: Scale-Related Face Validity Index; I-FVI: Item-Related Face Validity Index

Item	Rater 1	Rater 2	Rater 3	Rater 4	Rater 5	Rater 6	Rater 7	Rater 8	Rater 9	Rater 10	RA	I-FVI	UA
1	1	1	1	1	1	1	1	1	1	0	9	0.9	0
2	1	1	1	1	1	1	1	1	1	1	10	1	1
3	1	1	1	1	1	1	1	1	1	1	10	1	1
4	1	1	1	1	1	1	1	1`	1	1	10	1	1
5	1	1	1	1	1	1	1	1	0	1	9	0.9	0
6	1	1	1	1	1	1	1	1	1	0	10	0.9	0
7	1	1	1	1	1	1	1	1	1	1	10	1	1
8	1	1	1	1	1	1	1	1	1	1	10	1	1
9	1	1	1	1	1	1	1	1	1	1	10	1	1
10	1	1	1	1	1	1	1	1	1	1	10	1	1
11	1	1	1	1	1	1	1	1	1	1	10	1	1
12	1	1	1	1	1	1	1	1	1	0	9	0.9	0

**Table 3:** Content validity calculation R: Raters; CVR: Content Validity Ratio; I-CVI: Item-Related Content Validity Index; RA: Raters in Agreement; S-CVI: Scale-Related Content Validity Index

Item	R1	R2	R3	R4	R5	R6	R7	R8	R9	R10	CVR	I-CVI	RA	UA
First Round														
1	1	1	1	1	0	1	1	1	1	1	0.8	0.9	9	0
2	1	0	0	1	1	0	0	1	1	1	0.2	0.6	6	0
3	1	1	1	1	1	1	1	1	1	1	1	1	10	1
4	1	1	1	1	1	1	1	1	1	1	1	1	10	1
5	1	1	1	1	1	1	1	1	1	1	1	1	10	1
6	1	1	1	1	1	1	1	1	1	1	1	1	10	1
7	1	1	1	1	1	1	1	1	1	1	1	1	10	1
8	1	1	1	1	1	1	1	1	1	1	1	1	10	1
9	1	1	1	1	1	1	1	1	1	1	1	1	10	1
10	1	1	1	1	1	1	1	1	1	1	1	1	10	1
11	1	1	1	1	1	1	1	1	1	1	1	1	10	1
12	1	0	1	0	1	1	0	0	1	1	0.2	0.6	6	0
Proportion relevance	1	0.83	0.91	0.91	0.91	0.91	0.83	0.91	1	1	Ave CVR 0.85	S-CVI/Ave 0.92		S-CVI/UA 0.75
Second Round														
Item	R1	R2	R3	R4	R5	R6	R7	R8	R9	R10	CVR	I-CVI	RA	UA
1	1	1	1	1	1	1	1	1	1	1	1	1	10	1
2	1	1	1	1	1	0	1	1	1	1	0.8	0.9	9	0
3	1	1	1	1	1	1	1	1	1	1	1	1	10	1
4	1	1	1	1	1	1	1	1	1	1	1	1	10	1
5	1	1	1	1	1	1	1	1	1	1	1	1	10	1
6	1	1	1	1	1	1	1	1	1	1	1	1	10	1
7	1	1	1	1	1	1	1	1	1	1	1	1	10	1
8	1	1	1	1	1	1	1	1	1	1	1	1	10	1
9	1	1	1	1	1	1	1	1	1	1	1	1	10	1
10	1	1	1	1	1	1	1	1	1	1	1	1	10	1
11	1	1	1	1	1	1	1	1	1	1	1	1	10	1
12	1	1	1	1	1	1	1	1	1	1	1	1	10	1
Proportion relevance	1	1	1	1	1	0.91	1	1	1	1	Ave CVR 0.98	S-CVI/Ave 0.99		S-CVI/UA 0.91

Interobserver agreement

The analysis revealed differences in inter-observer reliability between the two assessment methods. For the traditional glance-and-grade method, the ICC was low (ICC = 0.129), indicating poor agreement between observers. The Pearson Correlation Coefficient also showed a weak, non-significant correlation (r = 0.131, p = 0.445). Similarly, the Wilcoxon Signed-Rank Test demonstrated no statistically significant difference between paired scores (p = 0.224). However, Bland-Altman analysis revealed a mean bias of 0.36, with limits of agreement ranging from -2.79 to 3.51, suggesting that, although the average scores were similar, discrepancies of up to approximately 3.5 marks could occur between observers (Table 4).

**Table 4:** Inter-examiner agreement analysis p-value < 0.05 is significant. ICC: Intraclass Correlation Coefficient; r: Pearson Correlation Coefficient

Test/Parameter	r (Pearson)	p-value	ICC (2,1)	ICC Category	Bland-Altman Mean Bias (SD)
Traditional: Observer 1 vs Observer 2 (Max score: 20)	0.131	0.445	0.129	Poor	0.361 (1.606)
New Method: Observer 3 vs Observer 4 (Max score: 15)	0.584	<0.001	0.526	Moderate	0.806 (1.766)

**Figure 2:**
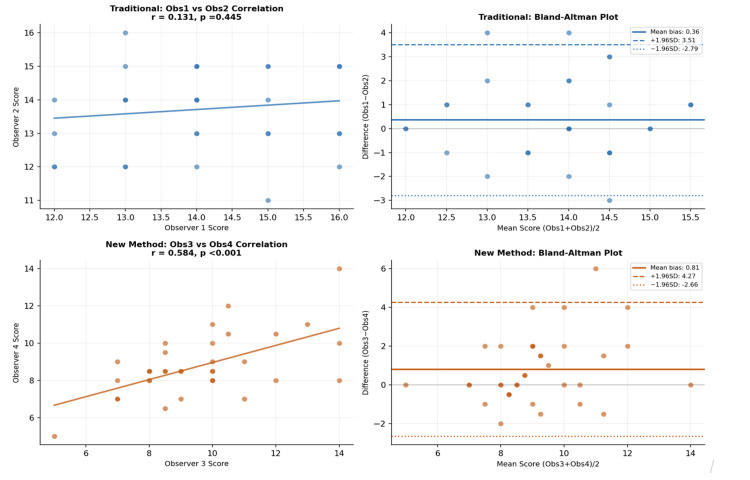
Inter-examiner agreement analysis

In contrast, the rubric-based assessment method demonstrated improved reliability. The ICC showed moderate agreement (ICC = 0.526), while the Pearson Correlation Coefficient indicated a statistically significant positive correlation between observers (r = 0.584, p < 0.001). The Wilcoxon Signed-Rank Test revealed a significant difference between the observers’ scores (p = 0.010), indicating that one examiner consistently awarded slightly higher marks than the other, highlighting the need for examiner calibration. Bland-Altman Analysis showed a mean bias of 0.81 between the two observers (Table 5 and Figure 2).

**Table 5:** Inter-examiner Wilcoxon Signed-Rank Test analysis W statistic: Wilcoxon Signed-Rank test statistic; New method: Rubric method; Traditional method: Glance-and-Grade method

Comparison	W statistic	p-value	Interpretation
Traditional: Obs1 vs Obs2 (inter)	129.0	0.224	No significant difference between observers - scores comparable
New Method: Obs3 vs Obs4 (inter)	74.0	0.010	Significant difference detected - observers score differently

Overall, the rubric-based method demonstrated superior inter-observer reliability compared with the traditional glance-and-grade approach (ICC = 0.526 vs. 0.129), although the level of agreement did not reach the threshold for good reliability (ICC > 0.75).

Intra-examiner reliability

The intra-examiner reliability analysis demonstrated differences in the consistency of scoring between the traditional and rubric-based assessment methods. For the traditional method, Observer 1 showed poor reliability, with a low ICC (ICC = 0.265). The Pearson Correlation Coefficient indicated a weak, non-significant correlation between the two assessments (r = 0.278, p = 0.101), and the Wilcoxon Signed-Rank Test showed no significant difference between the two scoring sessions (p = 0.399). The Bland-Altman Analysis revealed a small mean bias of 0.25, suggesting minimal systematic shift, but poor consistency in ranking individual students.

Observer 2, using the traditional method, also demonstrated poor reliability (ICC = 0.396), although a moderate and statistically significant correlation was observed (r = 0.545, p = 0.001). The Wilcoxon Signed-Rank Test showed a significant difference between the two assessments (p < 0.001), indicating that scores were systematically higher during the initial evaluation. The Bland-Altman Analysis indicated a mean bias of 1.14 marks, reflecting noticeable score drift over time.

In contrast, the rubric-based method demonstrated improved intra-examiner reliability. Observer 3 showed moderate reliability, with an ICC of 0.573 and a significant correlation between the two assessments (r = 0.573, p < 0.001). The Wilcoxon Signed-Rank Test revealed no significant difference between sessions (p = 0.604), and the Bland-Altman Analysis showed a negligible mean bias of -0.13, indicating stable scoring over time. Observer 4 also demonstrated moderate reliability (ICC = 0.600), with a significant correlation (r = 0.641, p < 0.001). Although the Wilcoxon Signed-Rank Test indicated a small but significant shift in scores (p = 0.005), the mean bias observed in the Bland-Altman Analysis was relatively modest (-0.67).

Overall, the rubric-based method produced consistently higher ICC values for intra-examiner reliability (0.573-0.600) compared with the traditional method (0.265-0.396). The traditional method demonstrated poorer consistency and greater score drift, particularly for Observer 2, whereas the rubric-based approach appeared to anchor scoring criteria and support more stable evaluations over time (Table 6).

**Table 6:** Intra-examiner agreement analysis ICC: Intraclass Correlation Coefficient; r: Pearson Correlation Coefficient; Wilcoxon p: Wilcoxon Signed-Rank Test; p < 0.05 is significant

Observer/Method	r (Pearson)	p-value	ICC (2, 1)	ICC Category	Wilcoxon p	Mean Bias (SD)
Observer 1 - Traditional (Max: 20)	0.278	0.101	0.265	Poor	0.399	0.25 (1.811)
Observer 2 - Traditional (Max: 20)	0.545	0.001	0.396	Poor	<0.001	1.14 (1.268)
Observer 3 - New Method (Max: 15)	0.573	<0.001	0.573	Moderate	0.604	-0.13 (1.845)
Observer 4 - New Method (Max: 15)	0.641	<0.001	0.600	Moderate	0.005	-0.67 (1.444)

**Figure 3:**
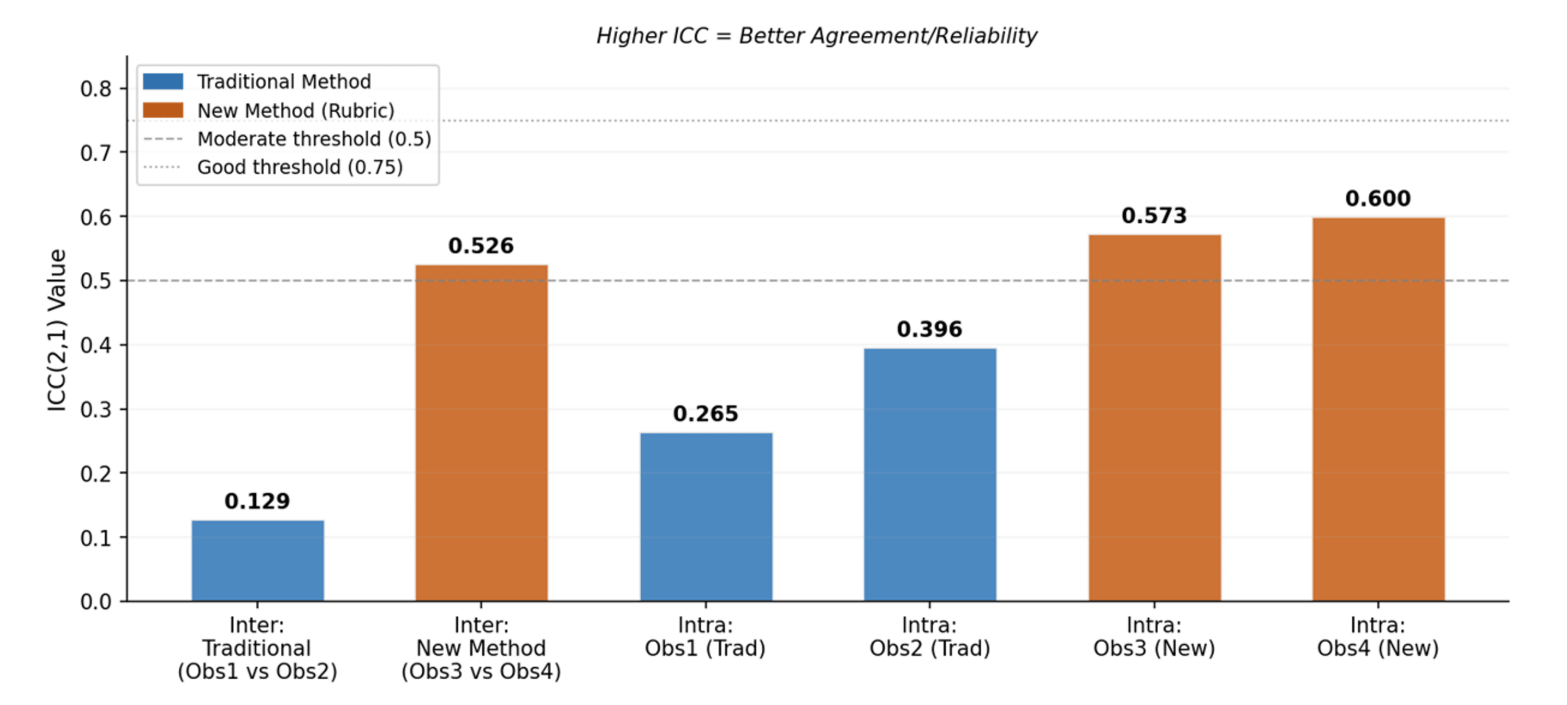
Bar chart of ICC values across all comparisons Dashed lines indicate moderate (0.5) and good (0.75) ICC thresholds. Orange bars (rubric method) consistently outperform blue bars (traditional glance-and-grade method). ICC: Intraclass Correlation Coefficient

Comparison of score levels between the two assessment methods was performed after converting the raw scores into percentages, to allow fair comparison (traditional method out of 20; rubric-based method out of 15). Using the Mann-Whitney U Test, the traditional glance-and-grade method demonstrated a significantly higher mean percentage score compared to the rubric-based method (69.51% vs 61.11%; U = 1017, p < 0.001). However, this higher score does not indicate greater reliability (Figure 3).

Instead, it likely reflects the inherent leniency associated with the traditional method, which may allow broader subjective judgment. In contrast, the rubric-based assessment applies predefined criteria, which may result in stricter, more discriminative scoring at the individual criterion level (Table 7).

**Table 7:** Summary of all statistical parameters ICC: Intraclass Correlation Coefficient

Parameter	Traditional (Glance & Grade)	New Method (Rubric)	Mann-Whitney U	p-value
Mean % Score (average of 2 observers)	69.51%	61.11%	1017.0	<0.001
Inter-observer ICC	0.129 (Poor)	0.526 (Moderate)	-	-
Intra-observer ICC (Obs A)	0.265 (Poor)	0.573 (Moderate)	-	-
Intra-observer ICC (Obs B)	0.396 (Poor)	0.600 (Moderate)	-	-
Intra-observer: Wilcoxon Obs A	p = 0.399 (NS)	p = 0.604 (NS)	-	-
Intra-observer: Wilcoxon Obs B	p < 0.001 (Sig)	p = 0.005 (Sig)	-	-

## Discussion

The present manuscript describes the development of a criteria-based rubric for evaluating pre-clinical competency in Class II cavity preparation on typodont teeth. An attempt was also made to test its reliability by evaluating inter- and intra-examiner agreement in scores using the glance-and-grade or rubric methods.

Evaluation of all domains of knowledge is a crucial part of medical education [12]. As this profession directly involves interactions with human beings and their organ systems, it becomes even more important that the education imparted to students is correctly conveyed, understood, and assimilated. Therefore, the tool used to evaluate cognitive or practical skills should be well-defined and able to provide feedback to students and teachers regarding progress in various aspects of competency. Here, the authors designed a rubric-based assessment tool and assessed its face validity and content validity to ascertain its aptness for use.

Face validity refers to the degree to which test respondents view the content of a test, and its items, as relevant to the context in which the test is being administered [14]. Because raters' backgrounds affect the interpretation of the tool, the assessment should be conducted by raters with a similar academic background or years of experience. Content validity, on the other hand, is defined as the degree to which elements of an assessment instrument are relevant to, and representative of, the targeted construct for a particular assessment purpose [15]. In the present study, we employed the 10 non-subject-matter experts method and the rubric method. However, inter- and intra-examiner variability persisted in their study's evaluation of content validity. The CVI and FVI were used to measure content validity and face validity, respectively. The FVI and CVI scores were 0.98 and 0.96, respectively, well above the minimum required to validate a tool. Researchers recommend that a scale with excellent content validity should be composed of I-CVIs of 0.78 or higher, and S-CVI/UA and S-CVI/Ave of 0.8 and 0.9 or higher, respectively [15].

The final rubric consisted of items related to instrument tray setup, initial cavity preparation, retention form, resistance form, manipulation of base and alloy/mercury, restoration of the cavity, and even handling of scrap amalgam. The items were assigned weights based on their importance within the competency. For example, setting up the instrument tray or handling scrap amalgam is important for the student to do correctly, but it is not a direct part of the tooth preparation process. Thus, these criteria were assigned a weight of 0.5, whereas steps such as retention form/resistance form were assigned a weight of 2 [16].

In the present study, a high level of consistency in scoring was observed among the examiners in the rubric group, whereas a fair level of agreement in scores was observed among the examiners of the glance-and-grade group. Inconsistency in results is one of the challenges faced when any assessment is made without a checklist, guide, or reference, and studies have been conducted to develop new, objective, and measurable assessment tools to overcome this difficulty.

In a study conducted by Escribano et al., inter-examiner agreement was evaluated for assessing portfolios of endodontic preclinical treatments performed by dental students using an analytic rubric and a numeric rating scale. They concluded that assessment guided by an analytic rubric allowed evaluators to reach higher levels of agreement than those obtained when using a numeric rating scale [17]. Sharaf et al. [11] conducted a study to evaluate the effectiveness of an analytic rubric for assessing pedodontic preclinical competencies on plastic primary teeth. In this study, the scoring of Class I, Class II, and Class V preparations was assessed using the glance-and-grade method, as well as the rubric method. However, inter-examiner and intra-examiner variability still existed in their study. The results of both studies suggested the presence of inconsistency among examiners when rating the preclinical performance of dental students. The inconsistent scores reflect subjectivity in the assessment methods, and this is one of the major complaints of the students - that there is no yardstick to measure why their scores, and those of their fellow students, vary. Lack of uniformity, uncertainty regarding importance, and lack of well-defined assessment criteria are some of the students’ more commonly expressed concerns [18].

One of the most important benefits of a well-designed rubric is that it facilitates learning by providing precise feedback to the students regarding specific areas that need improvement, and about what is being done well [19]. Usually, the conventional assessment methods leave the students with questions like where they went wrong, or what could have been done better. When given scores in a rubric format, they can review the score sheet and identify their areas of strength and weakness, thus providing a SWOC (Strengths, Weaknesses, Opportunities, Challenges) analysis of their performance.

The analytic rubric developed in the present study not only set levels of achievement against each criterion, but also defined weightage for each criterion. For example, the requirements of instrument tray setup, though an essential part of Class II tooth preparation, will carry less weight in the assessment of a Class II tooth preparation. This ensured that there was no over-deduction of marks for components that do not define the competency per se, but cannot be excluded from the checklists. This is in contrast to simple checklists of OSCE, where each point may carry an equal mark, and students who score better in less important components may also end up scoring a higher grade [20]. The final scores calculated for each criterion in the present analytic rubric were: weightage for that particular competency × marks for the level of achievement reached.

Apart from the students, rubrics can assist faculty members in upgrading their teaching skills. In a study conducted by Brown et al., where rubrics were used to assess compliance with aseptic techniques among pharmacy students, it was observed that not only were the errors among students decreased, but it also helped to recognize the weak points where the majority of students were scoring less, thus giving an idea about the need to adjust the curriculum and/or teaching methods accordingly [21].

However, there are specific challenges with using rubrics for assessment [22]. The prepared rubric should be lucid and understandable by both the student and the assessor. Care must be taken to ensure that the rubric encompasses all the essential components for that particular skill. Additionally, it should not be lengthy, as this may shift the focus from evaluation to page shuffling. In the present case, the final rubric encompassed all criteria, from instrument setup to the management of scrap amalgam after the restoration was complete. To overcome the length problem, the items up to tooth preparation were taken as one part, and from restoration onwards as another part. For the present study, only the assessment up to the tooth preparation part was conducted.

One limitation of the present study is that different examiner pairs were used to evaluate the traditional glance-and-grade method and the rubric-based assessment method. Consequently, the observed differences in reliability may partly reflect examiner-related variability, rather than the assessment tool alone. Future studies employing a crossover design, in which the same examiners evaluate both methods, would help better isolate the effect of the assessment approach.

## Conclusions

Based on the findings of the pilot study, the rubric-based assessment method is recommended as the primary tool for evaluating pre-clinical cavity preparations, as it demonstrated comparatively better inter- and intra-observer reliability than the traditional glance-and-grade approach. However, structured examiner calibration sessions should be conducted prior to routine implementation to further enhance scoring consistency and achieve higher reliability. The presence of statistically significant score shifts in certain observers indicates the possibility of score drift over time; therefore, periodic recalibration and refresher training for examiners are advisable. Future research should consider larger sample sizes and incorporate multiple calibration training iterations to determine whether the rubric-based assessment can achieve good reliability with experienced raters and further strengthen its applicability in pre-clinical dental education.
